# Molecular typing of *Trichomonas vaginalis* isolates by *actin* gene sequence analysis and carriage of *T. vaginalis* viruses

**DOI:** 10.1186/s13071-017-2496-7

**Published:** 2017-10-30

**Authors:** Simon C. Masha, Piet Cools, Tania Crucitti, Eduard J. Sanders, Mario Vaneechoutte

**Affiliations:** 1 Centre for Geographic Medicine Research – Coast, Kenya Medical Research Institute (KEMRI), P.O. Box 230-80108, Kilifi, Kenya; 2 Laboratory Bacteriology Research, Faculty of Medicine and Health Sciences, Ghent University, De Pintelaan, 185 9000 Ghent, Belgium; 3 Department of Biological Sciences, Faculty of Pure and Applied Sciences, Pwani University, P.O. BOX 195-80108, Kilifi, Kenya; 4 HIV/STI Reference Laboratory, Department of Clinical Sciences, Institute of Tropical Medicine, Nationalestraat 155, 2000 Antwerpen, Belgium

**Keywords:** *Trichomonas vaginalis*, *Trichomonas vaginalis* viruses, *actin* gene, Typing, Kilifi, Kenya

## Abstract

**Background:**

The protozoan parasite *Trichomonas vaginalis* is the most common non-viral, sexually transmitted pathogen. Although *T. vaginalis* is highly prevalent among women in Kenya, there is lack of data regarding genetic diversity of isolates currently in circulation in Kenya.

**Methods:**

Typing was performed on 22 clinical isolates of *T. vaginalis* collected from women attending the antenatal care clinic at Kilifi County Hospital, Kenya, in 2015. Genotyping followed a previously proposed restriction fragment length polymorphism (RFLP) scheme, which involved in silico cleavage of the amplified *actin* gene by *Hind*II, *Mse*I and *Rsa*I restriction enzymes. Phylogenetic analysis of all the sequences was performed to confirm the results obtained by RFLP-analysis and to assess the diversity within the RFLP genotypes. Additionally, we determined carriage of the four different types of *Trichomonas vaginalis* viruses (TVVs) by polymerase chain reaction.

**Results:**

In silico RFLP-analysis revealed five *actin* genotypes; 50.0% of the isolates were of *actin* genotype E, 27.3% of *actin* genotype N, 13.6% of *actin* genotype G and 4.5% of *actin* genotypes I and P. Phylogenetic analysis was in agreement with the RFLP-analysis, with the different *actin* genotypes clustering together. Prevalence of TVVs was 43.5% (95% confidence interval, CI: 23.2–65.5). TVV1 was the most prevalent, present in 39.1% of the strains and 90% of the *T. vaginalis* isolates which harbored TVVs had more than one type of TVV. None of the isolates of *actin* genotype E harbored any TVV.

**Conclusion:**

The presence of five *actin* genotypes in our study suggests notable diversity among *T. vaginalis* isolates occurring among pregnant women in Kilifi, Kenya. Isolates of the most prevalent *actin* genotype E lacked TVVs. We found no association between *T. vaginalis* genotype, carriage of TVVs and symptoms. Further studies with higher number of strains should be conducted in order to corroborate these results.

**Electronic supplementary material:**

The online version of this article (10.1186/s13071-017-2496-7) contains supplementary material, which is available to authorized users.

## Background

*Trichomonas vaginalis* is a flagellated protozoan parasite that infects the human urogenital tract, causing the most common non-viral, sexually transmitted infectious disease worldwide [1]. The prevalence of *T. vaginalis* among women in sub-Saharan Africa is 11.5% [1]. In about half of the infected women, *T. vaginalis* causes a malodorous vaginal discharge, vulval irritation and inflammation, and a ‘strawberry cervix’ characterized by punctate hemorrhagic lesions [2]. Men typically remain asymptomatic, but can suffer from urethral discharge, dysuria, urethritis, epididymitis and prostatitis [2, 3]. *Trichomonas vaginalis* has been associated with adverse pregnancy outcomes, such as preterm birth and premature rupture of membranes [4–6], increased shedding and acquisition of the human immunodeficiency virus (HIV) [7, 8], hence contributing to the HIV pandemic.

The global prevalence of *T. vaginalis* and the health sequelae associated with it have necessitated the need to understand its genetic make-up. *Trichomonas vaginalis* is a complex pathogen, with a genome size of ~160 megabases, two-thirds of which is composed of repeats and transposable elements [9]. Some strains of *T. vaginalis* can harbor up to four types of *Trichomonas vaginalis* viruses (TVVs) [10]. TVVs are members of the family *Totiviridae* under the distinct genus *Trichomonasvirus* [11]. Carriage of TVVs has been suggested to upregulate pro-inflammatory host responses [12] and *T. vaginalis* immunogenic protein P270 [13] and is also associated with differential qualitative and quantitative expression of cysteine proteinases [14]. Thus, since TVVs induce various phenotypic changes that may impact *T. vaginalis* virulence [14], determining carriage of TVVs seems to be essential in the characterization of *T. vaginalis* infection. However, no method has been adapted as a standard clinical diagnostic test for TVVs [15].

Better understanding the diversity of *T. vaginalis* and geographical distribution of various genotypes may improve our knowledge regarding the epidemiology of this infectious agent and contribute to vaccine development efforts. At present, none of the described techniques have been recognized as the “gold standard” for genotyping of *T. vaginalis* isolates [16–23].

In this study, we opted to sequence the *T. vaginalis actin* gene to better understand the genetic diversity of *T. vaginalis*. *Actin* is a ubiquitous well conserved structural protein in all eukaryotic cells [24] and has been used to clarify the molecular phylogeny of protists, plants, animals and fungi [25].

The data produced by sequencing are unambiguous, reproducible and portable, thus offering the advantage that public databases can be constructed. In Kenya, screening for *T. vaginalis* is not routinely done. However, recent studies have indicated that there is high prevalence of *T. vaginalis* among different groups in Kenya [26–29]. Despite this, no typing has been carried out. In this study, we genotyped *T. vaginalis* isolated from pregnant women attending ANC in Kilifi, Kenya.

## Methods

### Study design, population and specimens processing

From July through to September 2015, we conducted a cross-sectional study at the antenatal care clinic of Kilifi County Hospital, Kenya. The main aim of that study was to describe the prevalence and predictors of curable sexually transmitted infections (STIs) among pregnant women attending to the antenatal care clinic [29]. Women were eligible if they met the following criteria: age 18–45 years, gestation ≥ 14 weeks, resident of the Kilifi Health and Demographic Surveillance area, willingness to undergo free STI and bacterial vaginosis screening procedures, and able and willing to give written informed consent [29]. The current study presents a secondary objective of the above-mentioned study, namely to perform typing of *T. vaginalis* clinical isolates from this study population.

A total of 349 pregnant women were included in the study. A nurse collected vaginal secretions from the vaginal introitus using a sterile cotton swab. The vaginal swab was inoculated at the clinic in the upper-chamber of an InPouch system (BioMed Diagnostics, White City, Oregon, USA). The inoculated InPouch was transferred to the laboratory within 15 min for direct microscopy of the upper chamber, after which it was merged with the lower chamber and incubated at 37 ± 1 °C under aerobic conditions. Daily microscopic observation of the InPouch system was performed and media with motile trichomonads within 5 days of culture were considered positive for *T. vaginalis*. Two ml of the contents of each InPouch system positive for *T. vaginalis* were transferred into a 2.0 ml Eppendorf tube and stored at -80 °C until shipment to the Laboratory of Bacteriology Research (Ghent University, Belgium) using shipping boxes filled with dry ice (-78.5 °C). The samples were stored at -80 °C until molecular analysis was performed.

Women found to be positive for *T. vaginalis* using direct microscopy, i.e. microscopy of the upper chamber, were treated on the same day, while women who were negative for *T. vaginalis* using direct microscopy but positive on culture were contacted to return to the clinic for treatment immediately after the culture turned positive. Partners of women treated for *T. vaginalis* infection received presumptive treatment.

### Nucleic acid extraction

Frozen cultures were first thawed at room temperature for 30 min, after which the tubes were vortexed for 30 s to ensure that a homogeneous mix was achieved before starting the nucleic acid extraction. Total nucleic acid extraction was performed using the NucliSENS® easyMAG® (bioMérieux, Marcy l’Etoile, France) according to the manufacturer’s instructions (generic protocol 2.0.1).

### *Actin* gene PCR

The *actin* gene was amplified using the outer primers previously used in a nested polymerase chain reaction (PCR), i.e. primers Tv8S (5′-TCT GGA ATG GCT GAA GAA GAC G-3′) and Tv9R (5′-CAG GGT ACA TCG TAT TGG TC-3′) [20], with the following thermocycling conditions: 5 min at 95 °C, 40 cycles of 30 s at 95 °C, 30 s at 55 °C and 3 min at 72 °C, followed by 7 min at 72 °C. This was performed on the ABI Veriti thermocycler platform (ThermoFisher Scientific, Waltham, Massachusetts, USA).

### Gel electrophoresis

PCR amplification products were visualized under UV light after electrophoresis on 1% agarose gels in Tris-acetate-EDTA buffer pH 8.5 (30 min at 10 V/cm) and staining with ethidium bromide (0.5 mg⁄ l; Sigma, Bornem, Belgium). The size of the amplified products was assessed by comparison with a commercial weight marker, Smart Ladder (Eurogentec, Liege, Belgium).

### Sequencing

Amplicons (20 μl) were sent for sequencing to GATC Biotech (Constance, Germany), using the Sanger sequencing technique. Sequencing was performed bi-directionally using the same primers used in PCR amplification of the *actin* gene.

### Genotyping of *T. vaginalis*

*Trichomonas vaginalis actin* sequences were edited using Chromas Lite 2.01 (http://technelysium.com.au/wp/). Furthermore, we identified the *T. vaginalis actin* genotypes amongst our isolates by means of in silico RFLP-analysis Webcutter version 2.0; http://rna.lundberg.gu.se/cutter2/with the three restriction enzymes (*Hind*II, *Mse*I and *Rsa*I) used by Crucitti et al. [20]. The in silico analysis was done on our clinical isolates and on retrieved sequences representing the genotypes that have been proposed. To identify if there were additional *T. vaginalis actin* genotypes not captured by the previous proposed scheme, in silico RFLP was performed on *actin* sequences retrieved from the GenBank whose *actin* genotype is yet to be documented. The sequences were of at least 1100 bp in length (Additional file 1: Table S1).

To compare results obtained in the RFLP analysis, and to determine the genetic variation among the identified genotypes, we performed phylogenetic analysis of the sequences. Sequences obtained in our study were aligned using MEGA software and compared with analogous sequences representative of known *T. vaginalis actin* genotypes identified in from our isolates.

Evolutionary distances were calculated by Kimura’s two-parameter model (Kimura, 1980) and a phylogenetic tree was generated using the Maximum Likelihood method using the MEGA software (version 7.0) [30]. Finally, confidence levels were estimated using bootstrap resampling on 1000 randomly selected pseudoreplicates.

### Detection of *Trichomonas vaginalis* viruses

Synthesis of cDNA was performed on the nucleic acid extract using the RevertAid First Strand cDNA Synthesis Kit (ThermoFisher Scientific, Gent, Belgium). The cDNA was used to amplify the four known TVVs by means of previously described type specific primers: TVV1F2875 (5′-ATT AGC GGT GTT TGT GAT GCA-3′) and TVV1R3443 (5′-CTA TCT TGC CAT CCT GAC TC-3′), TVV2F1401 (5′-ATT AGC GGT GTT TGT GAT GCA-3′) and TVV2R1953b (5′-GGT TCG TGG AAG CGG TTG ATG A-3′), TVV3F1474 (5′-CTA CCA AGA AGG AGG CTT GA-3′) and TVV3R2025b (5′-GGT TCG TGG AAG CGG TTG ATG A-3′), and TVV4F1338 (5′-ATG CCA GTT GCT TTC CG-3′) and TVV4R1834 (5′-TTC CCC AAT AGT TAT CAG-3′) [10]. The amplification conditions were: 5 min at 95 °C, 40 cycles of 30 s at 95 °C, 30 s at 50 °C and 45 s at 72 °C, followed by 7 min at 72 °C. The PCR products were visualized by electrophoresis as described above.

### Statistical analysis

The socio-demographic and clinical data of the participants were entered in the REDCapTM electronic data capture tool, version 6.5.0 (Vanderbilt University, Nashville, Tennessee). Prevalence of *T. vaginalis* was expressed as a percentage with exact 95% binomial confidence intervals (CIs). Univariable logistic regression was used to determine associations of *T. vaginalis* presence with socio-demographic data, hygienic data, sexual behavior, and vaginal signs & symptoms. Variables significant at *P* ≤ 0.1 in bivariable analysis were entered into a multivariable model to identify independent associations. Crude odds ratios (COR) and adjusted odds ratios (AOR) were calculated. These statistical analyses were done using STATA, version 13.1 (Stata-Corp, College Station, Texas).

## Results

### Characteristics of the study population

The median age and gestation age including the interquartile range (IQR) for the participants was 27 (22–31) years and 25 (20–30) weeks, respectively. The majority of the participants were married (93.6%) and Christian (72.3%). Approximately a quarter of the participants had received secondary school education and above. The median age and IQR of sexual debut was 18.6 (16–20) years, although 17.4% of the participants were not sure or did not respond to this question. Seventy-three percent of the participants had given birth before and the number of children ranged between 1 and 10. Sixty-five percent of the participants had previously experienced signs/symptoms associated with reproductive tract infection and about one third had ever received syndromic treatment for genital signs or symptoms of infection. The socio-demographic characteristics of the participants are summarized in Table 1.

**Table 1:** Univariable and multivariable analysis of characteristics of pregnant women with *T. vaginalis* attending antenatal care clinic at Kilifi County Hospital, Kenya

Variable	*N* = 349	Percentage with *T. vaginalis*	Crude OR (95% CI)	*P*-value
Demographic characteristics				
Age group (years)				
18–24	132	6.8	1.1 (0.4–2.5)	0.894
≥ 25	217	6.5		
Religion				
Christian	248	5.7		
Muslim	54	5.7	1.0 (0.3–3.5)	0.979
Other/None	47	12.8	2.4 (0.9–6.7)	0.083^a^
Education				
None	63	7.9		
Primary	200	7.0	0.9 (0.3–2.5)	0.802
Secondary/Tertiary	86	4.7	0.6 (0.1–2.1)	0.411
Marital status				
Single	25	4.0		
Married	224	6.8	1.7 (0.2–13.5)	0.593
Residency				
Living with partner	255	5.5		
Not living with a partner	94	9.6	1.8 (0.8–4.4)	0.178
Employment status				
Employed/self-employed	199	5.0		
Unemployed	150	8.7	1.8 (0.8–4.2)	0.180
Parity				
0	94	6.4	1.1 (0.3–3.5)	0.843
1–2	133	7.5	1.3 (0.5–3.6)	0.570
3+	122	5.7		
Gestational age (weeks)^c^				
14–25	183	5.5		
≥ 26	165	7.9	1.5 (0.6–3.4)	0.382
Hygiene characteristics				
Toilet type				
Flushing toilet	117	7.7		
Pit latrine	197	5.1	0.6 (0.3–1.6)	0.350
Bush/Other	35	11.4	1.5 (0.4–5.4)	0.491
Mode of cleaning after visiting the toilet				
Tissue paper/Other solid materials	106	2.8		
Water	243	8.2	3.1 (0.9–10.5)	0.074^a^
Behavioral characteristics				
Sexual debut age (years)				
≤ 17	106	8.5	1.8 (0.7–4.7)	0.231
≥ 18	183	4.9		
Do not know/No response	60	6.7	1.5 (0.4–5.0)	0.523
Number of lifetime sex partners^c^				
1	158	3.8		
2+	189	9.0	2.5 (1.0–6.5)	0.060^a^
Polygamous partner				
No	303	6.9	1.6 (0.4–7.2)	0.515
Yes	46	4.4		
Alcohol consumption ever				
No	262	6.5	0.9 (0.4–2.5)	0.894
Yes	87	6.9		
Tobacco use				
No	332	6.6	1.1 (0.1–8.9)	0.904
Yes	17	5.9		
Other drugs/substance use ever				
No	332	6.6	1.1 (0.1–8.9)	0.904
Yes	17	5.9		
Sexually transmitted infections/reproductive tract infections				
HIV				
Negative	324	6.2		
Positive	25	12.0	2.1 (0.6–7.5)	0.268
Bacterial vaginosis^d^				
Negative	293	6.5		
Positive	53	7.6	1.1 (0.4–3.6)	0.775
Clinical signs and symptoms of STI				
Previous history of vaginal discharge				
No	122	5.6		
Yes	227	7.1	1.2 (0.5–3.1)	0.638
Previous syndromic treatment of genital infection				
No	229	6.6		
Yes	120	6.7	1.0 (0.4–2.5)	0.967
Current vaginal discharge (self-reported)^c^				
No	96	3.1		
Yes	252	7.9	2.7 (0.8–9.2)	0.119
Abnormal vaginal discharge foul smell/color (observed)^c^				
No	271	5.9		
Yes	77	9.1	1.6 (0.6–4.0)	0.324
Dysuria^c^				
No	263	6.1		
Yes	85	8.2	1.4 (0.5–3.5)	0.489
Dyspareunia^c^				
No	245	4.9		
Yes	103	10.7	2.3 (1.0–5.4)	0.053
Vaginal itching^c^				
No	231	6.5		
Yes	117	6.8	1.1 (0.4–2.6)	0.903
Lower abdominal pain^c^				
No	169	8.3		
Yes	179	5.0	0.6 (0.2–1.4)	0.226
Genital warts^c^				
No	341	6.2		
Yes	7	28.6	6.1 (1.1–33.3)	0.037^a^
Genital ulcer (observed)^c^				
No	338	5.6		
Yes	10	40.0	11.2 (2.9–43.1)	**< **0.001^b^
Vaginitis^c^				
No	334	6.6		
Yes	14	7.1	1.1 (0.1–8.7)	0.935
Symptomatic^c^				
No	145	6.9		
Yes	203	6.4	0.9 (0.4–2.2)	0.855

^a^Significant in univariable analysis

^b^Significant on multivariable association; symptomatic (any or a combination of the three symptoms, i.e. genital ulcer, lower abdominal pain or abnormal vaginal discharge)

^c^Missing data/some participants did not respond to this question(s)

^d^Bacterial vaginosis results for three participants were not available due to poor slides

### Prevalence, clinical signs and symptoms

A total of 23/349 (6.6%, 95% CI: 4.2–9.7) women were culture-positive for *T. vaginalis*, and 34.8% of these cases were positive by direct microscopy prior to further incubation. Based on symptoms routinely used in syndromic management of STIs (i.e. genital ulcer, lower abdominal pain or abnormal vaginal discharge), 43.5% of the 23 women with *T. vaginalis* infection were asymptomatic. The most commonly self-reported symptoms amongst all participants included vaginal discharge (72.4%). However, during collection of specimens by the study nurse, only 22.1% of the participants had an abnormal discharge (defined as excess discharge/foul smelling discharge/colored discharge) upon examination. A total of 51.4% of the women reported having lower abdominal pain, genital ulcers were observed in 2.9% of the women. Dyspareunia, genital warts and genital ulcers were the only clinical signs or symptoms significantly associated with *T. vaginalis* infection (Chi-square test: *χ* ^2^ = 3.93, *df* = 1, *P* < 0.048; *χ* ^2^ = 5.58, *df* = 1, *P* < 0.018; and *χ* ^2^ = 18.60, *df* = 1, *P* < 0.05, respectively).

### Predictors of *T. vaginalis* infection

Univariable analysis indicated that *T. vaginalis* infection was more common among participants who were traditionalist or reported having no religion compared to participants who were Christians or Muslims, used water to clean themselves after visiting the toilet compared to those who used tissue paper or other solid materials, reported having ≥ 2 lifetime sexual partners, reported dyspareunia, had genital warts, and/or had a genital ulcer (Table 1). In multivariable analysis, the only independent predictor associated with *T. vaginalis* was having a genital ulcer (AOR = 7.6, 95% CI: 1.4–42.3).

### Genotyping analysis

The *actin* gene target could be amplified from 21 of the 23 *T. vaginalis* isolates. All 21 amplicons had the expected length of approximately 1100 bp. The two remaining isolates could only be amplified by a higher primer concentration of 0.5 μM, instead of 0.3 μM. However, one of these clinical isolates did not yield an interpretable sequence and thus only sequences from 22 clinical isolates were utilized in the typing. Five different *actin* types (E, G, I, N and P) were identified according to the position and the number of cleavage sites, following the scheme proposed by Crucitti et al. [20] (Table 2). The most prevalent *actin* genotype was E, representing 50.0% of the isolates. The other genotypes were, in order of descending frequency, N (27.3%), G (13.6%) and I and P (each 4.5%).

**Table 2:** Number and position of restriction sites using *Hind*II, *Mse*I and *Rsa*I restriction enzymes

Genotype	No. of isolates	*Hind*II			*Mse*I			*Rsa*I				
		213	273	699	185	314	518	103	190	426	878	994
E	11	×	×			×	×	×	×	×		×
G	3	×	×	×			×		×	×		×
I	1	×	×	×			×		×	×	×	×
N	6	×	×	×	×		×	×	×	×		×
P	1	×	×	×			×	×	×	×	×	×

× indicates the presence of a restriction cut site

**Fig. 1:**
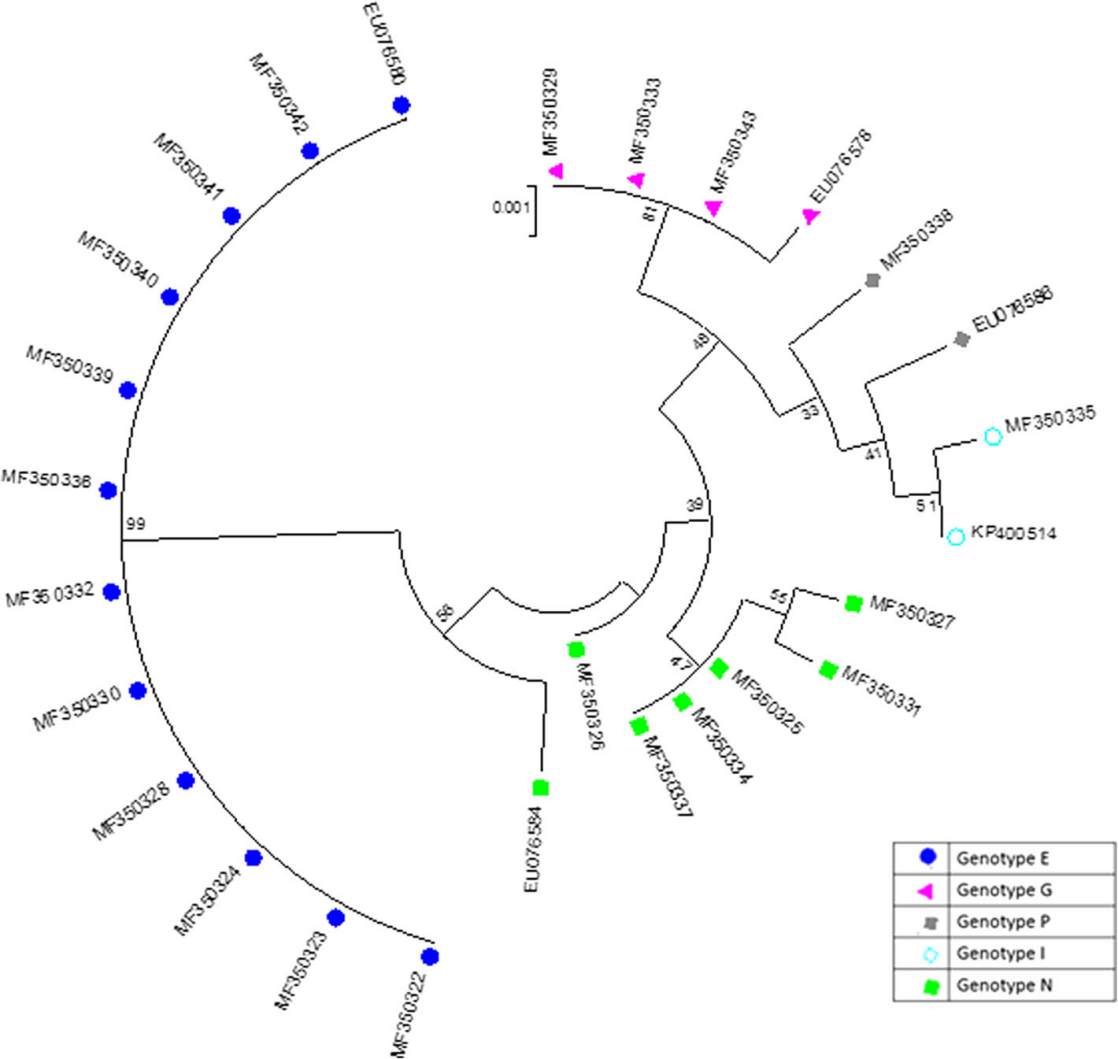
Phylogenetic tree for *actin* gene nuclotide sequences of *T. vaginalis*. The tree was generated using Maximum Likelihood method. Bootstrap test for 1000 replicates. Sequences from Kilifi isolates (*n* = 22) have accession numbers with the prefix “MF”, all other sequences were retreived from GenBank and are representative of the five actin genotypes. *Scale-bar*: 0.001 (1 substitution per 1000 nucleotides)

Multiple sequence analysis to compare polymorphic sites found on our *actin* sequences, and those retrieved from the GenBank, revealed a total of 33 single nucleotide differences in the open reading frame of the *actin* gene. Three of these single nucleotide polymorphisms were exclusively found in *actin* sequences from our study (Additional file 2). The nucleotide sequences obtained of the *actin* gene for all the 22 isolates were submitted in GenBank under accession numbers: (MF350322–MF350343). The phylogenetic analysis (Fig. 1) showed that *actin* genotype E clustered with a bootstrap value of 99. Lower bootstrap values were observed for *actin* genotypes N, G, I and P.

### Prevalence of *T. vaginalis* viruses

TVVs were present in 43.5% (95% CI: 23.2–65.5) (10/23) of *T. vaginalis* isolates. *Trichomonas vaginalis* virus type 1 (TVV1) was the most prevalent (39.1%), followed by TVV2 (26.1%), TVV3 (17.4%) and TVV4 (13.0%). Nine out of 10 *T. vaginalis* isolates with TVVs harbored more than one type of TVV (Table 3). TVV1 was present in all virus-positive *T. vaginalis* isolates but one (TV207). All 11 genotype E isolates were virus-negative.

**Table 3:** Genotypes of *Trichomonas vaginalis* and carriage of *T. vaginalis* viruses, in relation to symptoms among 23 *T. vaginalis* isolates in Kilifi, Kenya

Sample ID	TVV1	TVV2	TVV3	TVV4	Genotype	Symptomatic^a^
TV279	+	–	+	–	*	+
TV022	–	–	–	–	E	+
TV042	–	–	–	–	E	+
TV050	–	–	–	–	E	–
TV066	–	–	–	–	E	+
TV075	–	–	–	–	E	+
TV176	–	–	–	–	E	–
TV188	–	–	–	–	E	–
TV203	–	–	–	–	E	+
TV224	–	–	–	–	E	–
TV299	–	–	–	–	E	+
TV323	–	–	–	–	E	–
TV185	+	–	+	+	G	–
TV207	–	+	–	–	G	+
TV238	+	+	+	–	G	+
TV307	+	+	–	–	I	–
TV116	+	+	–	–	N	–
TV131	+	+	–	–	N	+
TV156	+	–	–	+	N	–
TV190	+	+	+	–	N	+
TV210	–	–	–	–	N	+
TV234	+	–	–	+	N	–
TV140	–	–	–	–	P	+

*Abbreviations*: *TVV1 Trichomonas vaginalis* virus type 1, *TVV2 T. vaginalis* virus type 2, *TVV3 T. vaginalis* virus type 3, *TVV4 T. vaginalis* virus type 4

*Code*: *Not typed; + present; − absent

^a^Symptomatic: any or a combination of the three symptoms, i.e. genital ulcer, lower abdominal pain and/or abnormal vaginal discharge

Finally, the distribution of symptomatic and asymptomatic cases was not linked to any particular *T. vaginalis actin* genotype. Similarly, presence or absence of TVVs did not appear to have an influence as to whether a patient was symptomatic or asymptomatic.

## Discussion

To our knowledge, this is the first study determining *T. vaginalis* genotypes and co-occurrence of *T. vaginalis* viruses in Kenya. We sequenced the *actin* gene for 22 isolates and identified five types of *T. vaginalis* by in silico RFLP-analysis of the amplified *actin* gene. We found notable genetic diversity by full *actin* gene sequence analysis among *T. vaginalis* isolates in Kilifi, as well as those retrieved from GenBank. Prevalence of *T. vaginalis* in this study (6.6% in 349 pregnant women) was high but, however, fell short of the prevalence of *T. vaginalis* among women aged 15–49 years in the World Health Organization Africa region in 2012, which was estimated to be 11.5% (95% CI: 9.0–14.6) [1]. This is suggestive of lower rates of *T. vaginalis* among the general population of women in Kilifi, Kenya compared to other African countries [31].

Although *T. vaginalis* does not traditionally present with genital ulcers [32], multivariable analysis showed that genital ulcers were the only predictor of an infection with *T. vaginalis* in our study. Ulcers could not be due to syphilis, which was not diagnosed in any of the women with *T. vaginalis*, but we did not test for the Herpes simplex virus, which is also associated with genital ulcers. The association of genital ulcer with *T. vaginalis* is not unique to our study as it has been reported amongst female sex workers in China [33].

Nucleotide sequence analysis of *actin* sequences showed 33 polymorphic sites, three of which caused amino acid substitution. Two of these amino acid substitutions have been previously reported to occur in genotypes G, N, I and P, in which nucleotide 371 substituted alanine for valine, and nucleotide 904 substituted lysine for glutamine [20, 34]. A unique polymorphism leading to an amino acid substitution, in which nucleotide 892 substituted threonine for serine, was observed to be exclusively present on a GenBank sequence (accession number XM_001301892). As such, in silico genotyping of isolates provides an opportunity to distinguish closely related isolates based on these polymorphic sites and to further identify such polymorphic sites.

Our phylogenetic analysis confirms RFLP as a good typing method, as the results from this method were in agreement with phylogenetic analysis. Phylogenetic analysis and detection of carriage of TVVs, revealed that none of the isolates of the most prevalent *actin* genotype E harbored a TVV. Furthermore, phylogenetic analysis indicated that genotype E formed a distinct phylogenetic lineage, suggesting clonal stability of this genotype [35].

The high prevalence (43.5%) of TVVs found in this study is comparable to a prevalence of 55% (95% CI: 38.4–70.7) among Cuban isolates [36]. Two recent studies have reported lower carriage of TVV, 18.7% (95% CI: 11.5–28.0) in the Philippines [37] and 17.3% (95% CI: 7.8–31.4) among Iranian isolates [38], although the latter study only determined the presence of TVV1. However, higher prevalence rates of TVV have been reported as well, 81.9% (95% CI: 71.1–90.0) in South Africa [39], and 75.0% (95% CI: 55.1–89.3) in Baltimore City, Maryland [40]. The presence of TVV, in addition to metronidazole susceptibility, has been found to differ significantly between *T. vaginalis* isolates genotyped by a panel of 21 microsatellites and six single-copy genes of *T. vaginalis*, which classifies *T. vaginalis* into two types: type 1 and type 2 [23]. Type 2 is characteristically free of TVV and resistant to metronidazole [23]. Metronidazole susceptibility in relation to *actin* genotypes is yet to be determined.

Fifty-seven percent of isolates in our study did not harbor TVV, suggesting that they might be of type 2. Our study might have been biased towards type 2; 63.6% were recovered from patients who were diagnosed by culture, after direct microscopy had been determined to be negative, suggesting that the parasite load in the patient was low. Conrad et al. [23] observed that type 1 parasites are often diagnosed by direct microscopy and suggested that this may be indicative of higher parasite load in type 1 which harbor TVVs. Additional studies, sampling a more diverse population and other regions in Kenya, are needed to confirm the population type and distribution of *T. vaginalis* in the country.

A total of 22 TVVs were identified in 10 *T. vaginalis* cultures, with multiple TVVs detected in nine cultures (Table 2). The higher prevalence of *T. vaginalis* cultures with either TVV1 or TVV2 than with TVV3 or TVV4 is consistent with previous reports [10, 37]. In these publications, concurrent TVV infection, with at least two or 3 TVVs, was recorded in six and three *T. vaginalis* sample cultures, respectively. Although we identified single *actin* genotypes in all *T. vaginalis* cultures, which is indicative for the presence of only 1 *T. vaginalis* strain per culture, we cannot rule out that the presence of the multiple TVVs may be the result of a mixture of *T. vaginalis* strains, each infected with a different TVV. Therefore, our data does not necessarily indicate concurrent infection of TVVs in a single TV strain. The lytic cycle of TVVs is yet to be described, and attempts to infect uninfected isolates have been unsuccessful [41]. Therefore, it is plausible that the virus may solely be acquired through vertical transmission, making its presence an important genetic marker [42].

Fifty-six percent of women with *T. vaginalis* infection in our study were symptomatic and the symptoms occurred independently of the presence of TVV in the protozoon. In vitro studies have shown that TVVs are sensed by the human epithelial cells via Toll-like receptor 3, triggering Interferon Regulating Factor − 3, interferon type I and pro-inflammatory cascades previously implicated in preterm birth and HIV-1 susceptibility [12]. While treatment with metronidazole generally eliminates *T. vaginalis*, this may aggravate *T. vaginalis*-associated inflammation caused by the release of TVV by stressed or dying parasites [12]. Additionally, TVVs upregulate levels of phenotypically variable immunogen mRNA P270 of *T. vaginalis* [13], while also playing a role in *T. vaginalis* protein composition and its growth kinetics [14].

## Conclusion

Our study was limited by the small number of isolates, which rendered it difficult to investigate the implication of TVV carriage on clinical signs and symptoms. Despite the low number of *T. vaginalis* isolates, the presence of all four types of TVVs in our isolates, in addition to the five *actin* genotypes, demonstrates there is notable genetic diversity of *T. vaginalis* isolated from pregnant women in Kilifi, Kenya. Isolates of the most prevalent *actin* genotype E lacked TVVs; further studies with higher number of strains should be conducted in order to corroborate these results. The *actin* gene should be considered as a potential genetic marker for molecular epidemiology and genotypic traits of *T. vaginalis*.

## Additional files

Additional file 1: Table S1. Genotype, number and position of restriction sites using *Hind*II, *Mse*I and *Rsa*I restriction enzymes for *actin* sequences retrieved from GenBank. (DOCX 15 kb)

Additional file 2: Figure S1. Alignment of the *T. vaginalis actin* gene nucleotide sequences retrieved from GenBank and those of from the clinical *T. vaginalis* isolates of the present study. (PDF 1746 kb)

## Abbreviations

RFLP: Restriction fragment length polymorphism
TVV: *Trichomonas vaginalis* viruses
