# Supplementary material for: Molecular typing of Trichomonas vaginalis isolates by actin gene sequence analysis and carriage of T. vaginalis viruses
Source: Parasit Vectors. 2017 Oct 30;10:537. doi: 10.1186/s13071-017-2496-7 (PMC5663105; doi:10.1186/s13071-017-2496-7)
Supplement: Supplementary file 1 — Genotype, number and position of restriction sites using HindII, MseI and RsaI restriction enzymes for actin sequences retrieved from GenBank. (DOCX 15 kb) [file 13071_2017_2496_MOESM1_ESM.docx]

**Additional file 1: Table S1.** Genotype, number and position of restriction sites using *Hind*II, *Mse*1 and *Rsa*1 restriction enzymes for actin sequences retrieved from GenBank

| **Gene Bank Accession** | **Origin** | **Position of restriction sites** | | | **Genotype** |
| --- | --- | --- | --- | --- | --- |
|  |  | **HindII** | **MseI** | **RsaI** |  |
| KX452108 | Iran | 213 273 | 314 518 | 103 190 426 994 | E |
| KX452109 | Iran | 213 273 | 314 518 | 103 190 426 994 | E |
| KX452110 | Iran | 213 273 | 314 518 | 103 190 426 994 | E |
| KX452111 | Iran | 213 273 | 314 518 | 103 190 426 994 | E |
| EU0765780 | ATCC 50141 | 213 273 | 314 518 | 103 190 426 994 | E |
| KF747375 | Iran | 213 273 | 314 518 | 103 190 426 994 | E |
| KF747376 | Iran | 213 273 | 314 518 | 103 190 426 994 | E |
| KP400515 | Iran | 213 273 | 314 518 | 103 190 426 994 | E |
| KP400516 | Iran | 213 273 | 314 518 | 103 190 426 994 | E |
| EU076582 | Zambia | 213 273 | 314 518 | 103 190 426 994 | E |
| EU076578 | ATCC 30001 | 213 273 699 | 518 | 190 426 994 | G |
| KF747377 | Iran | 213 273 699 | 518 | 190 426 994 | G |
| XM_001301892.1 | strain PRA-98 (G3) | 213 273 699 | 518 | 103 190 426 994 | H |
| EU076579 | ATCC 30240 | 213 273 699 | 518 | 103 190 426 994 | H |
| EU0765781 | Zambia | 213 273 699 | 518 | 103 190 426 994 | H |
| KP400513 | Iran | 213 273 699 | 518 | 103 190 426 994 | H |
| EU076585 | Zambia | 213 273 699 | 518 | 190 426 878 994 | I |
| EU076583 | DRC | 213 273 699 | 185 518 | 190 426 994 | M |
| EU076584 | DRC | 213 273 699 | 185 518 | 103 190 426 994 | N |
| EU076586 | Zambia | 213 273 699 | 518 | 103 190 426 878 994 | P |
| KP400514 | Iran | 213 273 699 | 518 | 103 190 426 878 994 | P |

DRC Democratic Republic of the Congo
