# Supplementary material for: Molecular typing of Trichomonas vaginalis isolates by actin gene sequence analysis and carriage of T. vaginalis viruses
Source: Parasit Vectors. 2017 Oct 30;10:537. doi: 10.1186/s13071-017-2496-7 (PMC5663105; doi:10.1186/s13071-017-2496-7)
Supplement: Supplementary file 2 — Alignment of the T. vaginalis actin gene nucleotide sequences retrieved from GenBank and those of from the clinical T. vaginalis isolates of the present study. (PDF 1746 kb) [file 13071_2017_2496_MOESM2_ESM.pdf]

Additional file 2: Alignment of the *T. vaginalis* actin gene nucleotide sequences retrieved from GenBank and those of from our clinical *T. vaginalis* isolates

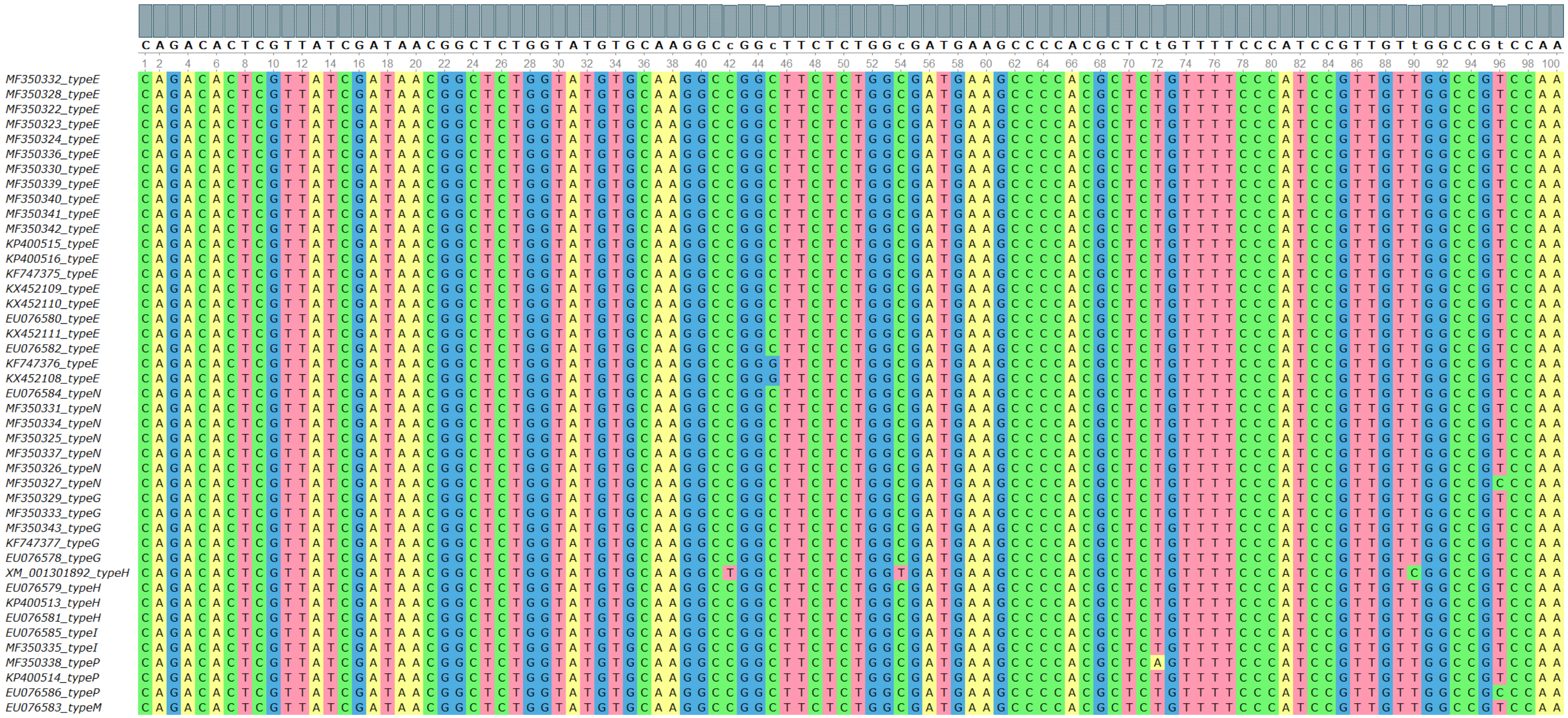

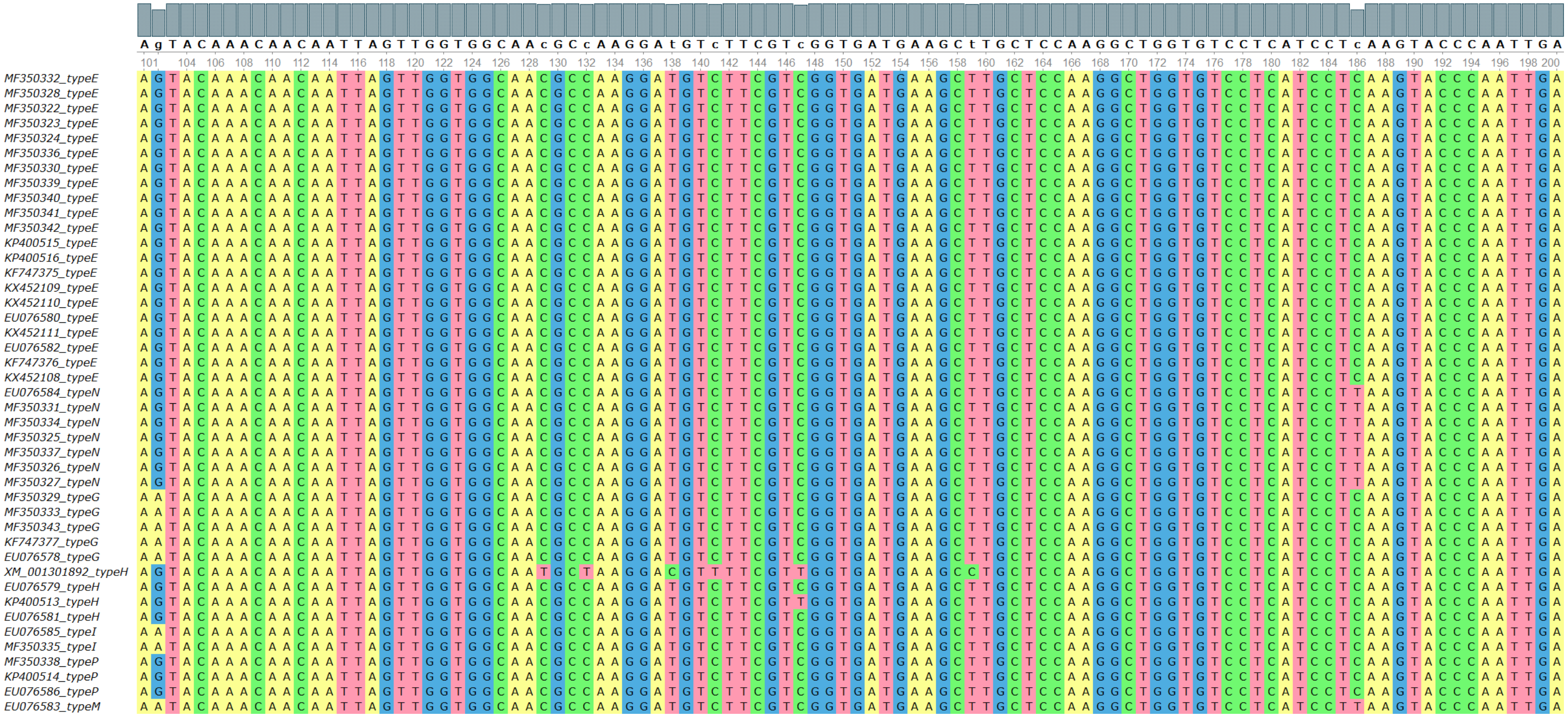
AgTACAAACAACAATTAGTTGGTGGCAAcGCcAAGGAtGTcTTCGTcGGTGATGAAGCtTGCTCCAAGGCTGGTGTCCTCATCCTcAAGTACCCAATTGA
101 104 106 108 110 112 114 116 118 120 122 124 126 128 130 132 134 136 138 140 142 144 146 148 150 152 154 156 158 160 162 164 166 168 170 172 174 176 178 180 182 184 186 188 190 192 194 196 198 200
MF350332_typeE
MF350328_typeE
MF350322_typeE
MF350323_typeE
MF350324_typeE
MF350336_typeE
MF350330_typeE
MF350339_typeE
MF350340_typeE
MF350341_typeE
MF350342_typeE
KP400515_typeE
KP400516_typeE
KF747375_typeE
KX452109_typeE
KX452110_typeE
EU076580_typeE
KX452111_typeE
EU076582_typeE
KF747376_typeE
KX452108_typeE
EU076584_typeN
MF350331_typeN
MF350334_typeN
MF350325_typeN
MF350337_typeN
MF350326_typeN
MF350327_typeN
MF350329_typeG
MF350333_typeG
MF350343_typeG
KF747377_typeG
EU076578_typeG
XM_001301892_typeH
EU076579_typeH
KP400513_typeH
EU076581_typeH
EU076585_typeI
MF350335_typeI
MF350338_typeP
KP400514_typeP
EU076586_typeP
EU076583_typeM

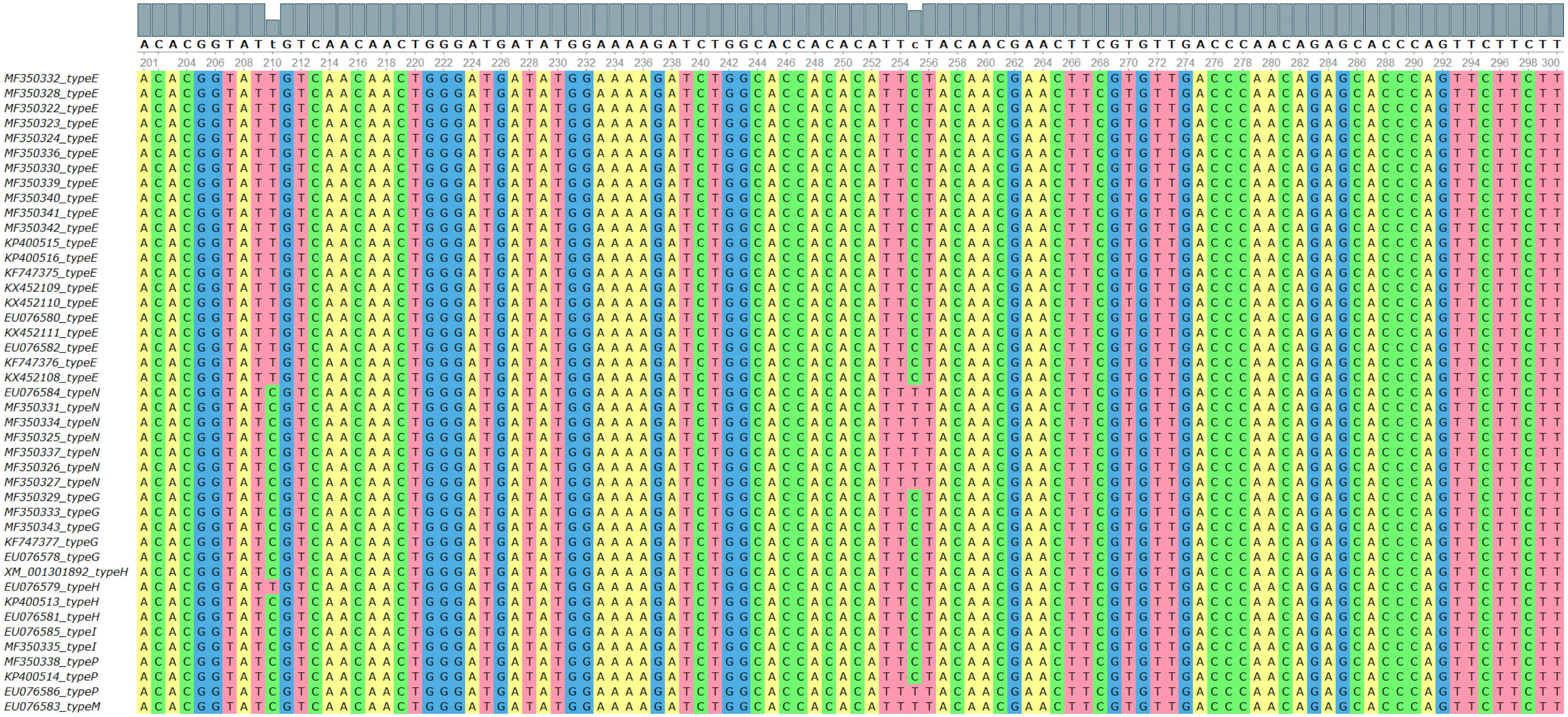
ACACGGTATtGTCAACAACTGGGATGATATGGAAAAGATCTGGCACCACACATTcTACAACGAACTTCGTGTTGACCCAACAGAGCACCCAGTTCTTCTT
201 204 206 208 210 212 214 216 218 220 222 224 226 228 230 232 234 236 238 240 242 244 246 248 250 252 254 256 258 260 262 264 266 268 270 272 274 276 278 280 282 284 286 288 290 292 294 296 298 300
MF350332_typeE
MF350328_typeE
MF350322_typeE
MF350323_typeE
MF350324_typeE
MF350336_typeE
MF350330_typeE
MF350339_typeE
MF350340_typeE
MF350341_typeE
MF350342_typeE
KP400515_typeE
KP400516_typeE
KF747375_typeE
KX452109_typeE
KX452110_typeE
EU076580_typeE
KX452111_typeE
EU076582_typeE
KF747376_typeE
KX452108_typeE
EU076584_typeN
MF350331_typeN
MF350334_typeN
MF350325_typeN
MF350337_typeN
MF350326_typeN
MF350327_typeN
MF350329_typeG
MF350333_typeG
MF350343_typeG
KF747377_typeG
EU076578_typeG
XM_001301892_typeH
EU076579_typeH
KP400513_typeH
EU076581_typeH
EU076585_typeI
MF350335_typeI
MF350338_typeP
KP400514_typeP
EU076586_typeP
EU076583_typeM

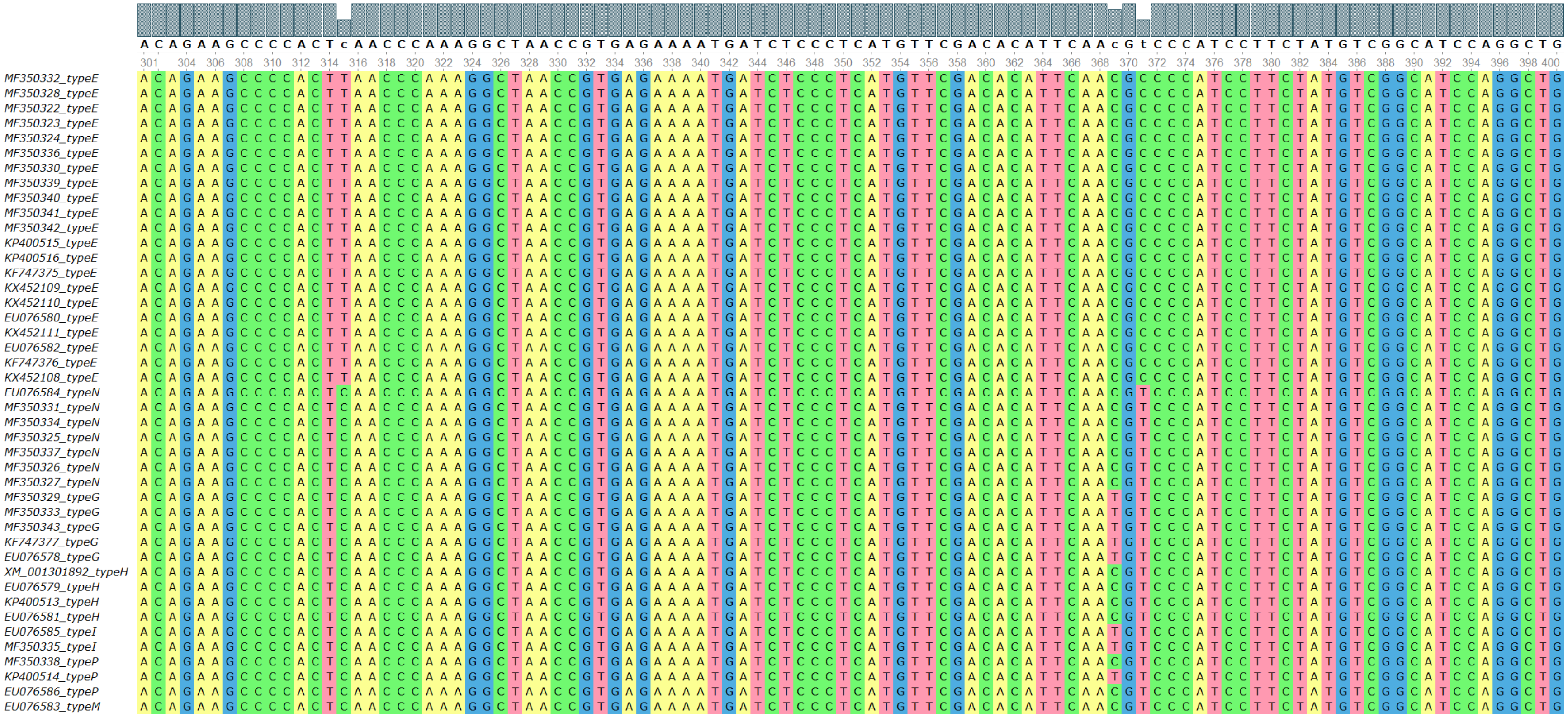
A C A G A A G C C C C A C T c A A C C C A A A G G C T A A C C G T G A G A A A A T G A T C T C C C T C A T G T T C G A C A C A T T C A A c G t C C C A T C C T T C T A T G T C G G C A T C C A G G C T G
301 304 306 308 310 312 314 316 318 320 322 324 326 328 330 332 334 336 338 340 342 344 346 348 350 352 354 356 358 360 362 364 366 368 370 372 374 376 378 380 382 384 386 388 390 392 394 396 398 400
MF350332_typeE
MF350328_typeE
MF350322_typeE
MF350323_typeE
MF350324_typeE
MF350336_typeE
MF350330_typeE
MF350339_typeE
MF350340_typeE
MF350341_typeE
MF350342_typeE
KP400515_typeE
KP400516_typeE
KF747375_typeE
KX452109_typeE
KX452110_typeE
EU076580_typeE
KX452111_typeE
EU076582_typeE
KF747376_typeE
KX452108_typeE
EU076584_typeN
MF350331_typeN
MF350334_typeN
MF350325_typeN
MF350337_typeN
MF350326_typeN
MF350327_typeN
MF350329_typeG
MF350333_typeG
MF350343_typeG
KF747377_typeG
EU076578_typeG
XM_001301892_typeH
EU076579_typeH
KP400513_typeH
EU076581_typeH
EU076585_typeI
MF350335_typeI
MF350338_typeP
KP400514_typeP
EU076586_typeP
EU076583_typeM

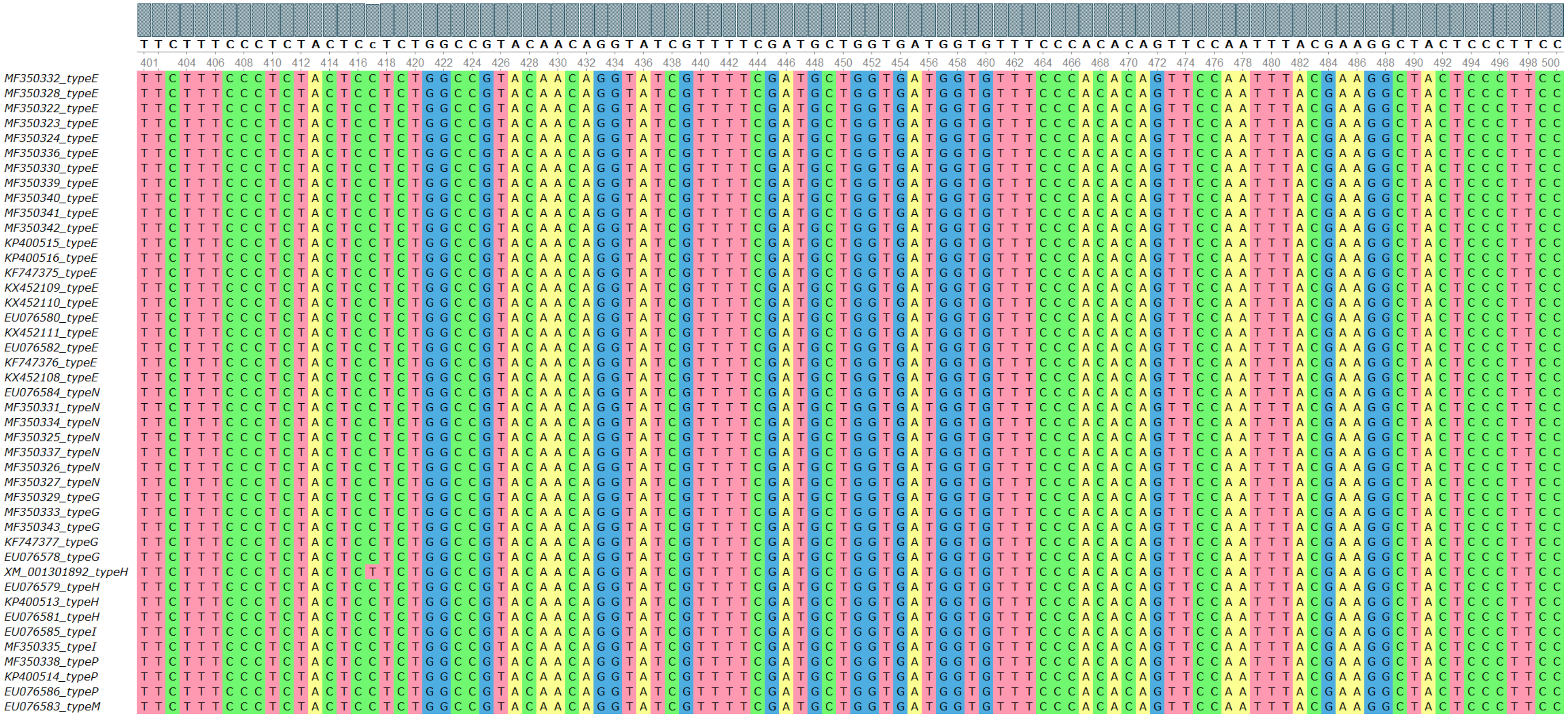
MF350332_typeE
MF350328_typeE
MF350322_typeE
MF350323_typeE
MF350324_typeE
MF350336_typeE
MF350330_typeE
MF350339_typeE
MF350340_typeE
MF350341_typeE
MF350342_typeE
KP400515_typeE
KP400516_typeE
KF747375_typeE
KX452109_typeE
KX452110_typeE
EU076580_typeE
KX452111_typeE
EU076582_typeE
KF747376_typeE
KX452108_typeE
EU076584_typeN
MF350331_typeN
MF350334_typeN
MF350325_typeN
MF350337_typeN
MF350326_typeN
MF350327_typeN
MF350329_typeG
MF350333_typeG
MF350343_typeG
KF747377_typeG
EU076578_typeG
XM_001301892_typeH
EU076579_typeH
KP400513_typeH
EU076581_typeH
EU076585_typeI
MF350335_typeI
MF350338_typeP
KP400514_typeP
EU076586_typeP
EU076583_typeM

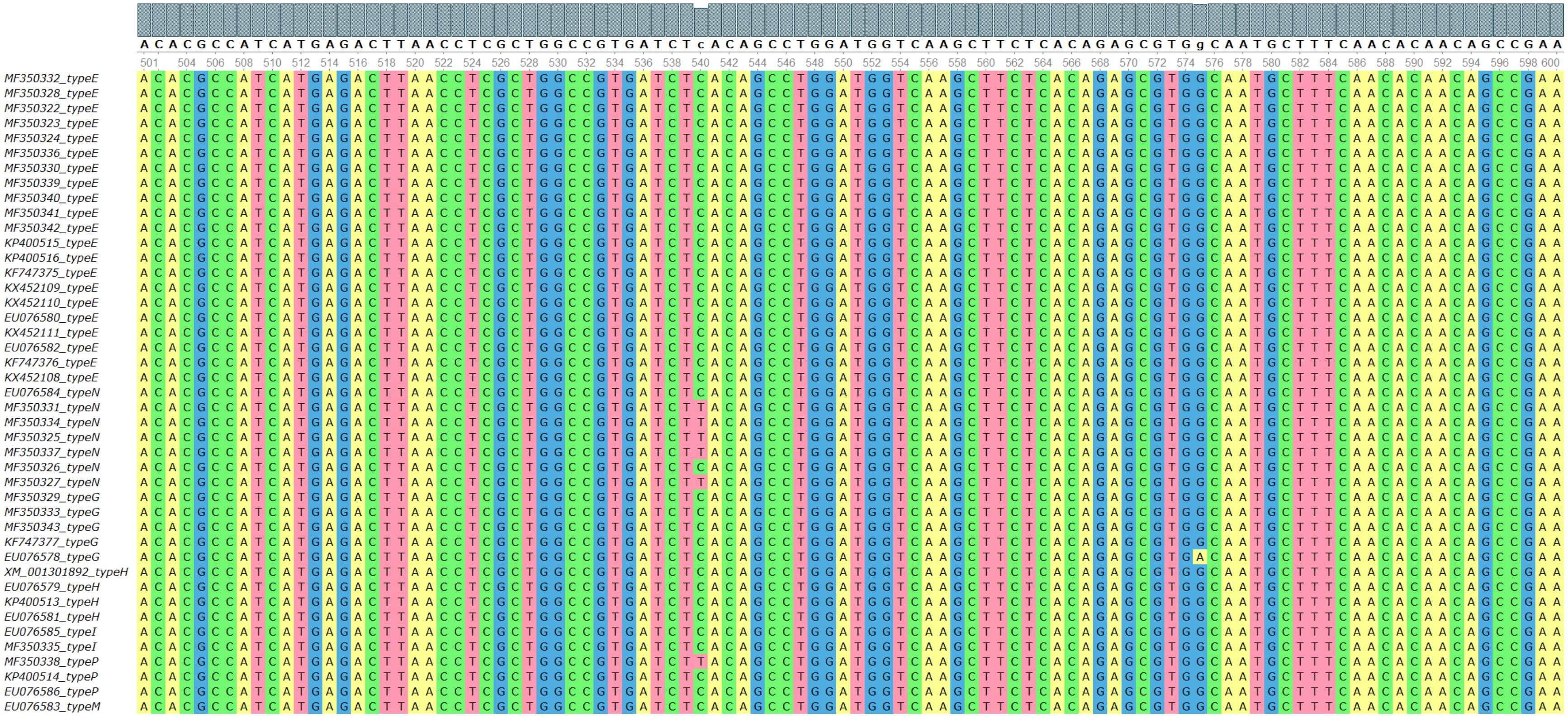

ACACGCCATCATGAGACTTAACCTCGCTGGCCGTGATCTcACAGCCTGGATGGTCAAGCTTCTCACAGAGCGTGgCAATGCTTTCAACACAACAGCCGAA
501 504 506 508 510 512 514 516 518 520 522 524 526 528 530 532 534 536 538 540 542 544 546 548 550 552 554 556 558 560 562 564 566 568 570 572 574 576 578 580 582 584 586 588 590 592 594 596 598 600
MF350332_typeE
MF350328_typeE
MF350322_typeE
MF350323_typeE
MF350324_typeE
MF350336_typeE
MF350330_typeE
MF350339_typeE
MF350340_typeE
MF350341_typeE
MF350342_typeE
KP400515_typeE
KP400516_typeE
KF747375_typeE
KX452109_typeE
KX452110_typeE
EU076580_typeE
KX452111_typeE
EU076582_typeE
KF747376_typeE
KX452108_typeE
EU076584_typeN
MF350331_typeN
MF350334_typeN
MF350325_typeN
MF350337_typeN
MF350326_typeN
MF350327_typeN
MF350329_typeG
MF350333_typeG
MF350343_typeG
KF747377_typeG
EU076578_typeG
XM_001301892_typeH
EU076579_typeH
KP400513_typeH
EU076581_typeH
EU076585_typeI
MF350335_typeI
MF350338_typeP
KP400514_typeP
EU076586_typeP
EU076583_typeM

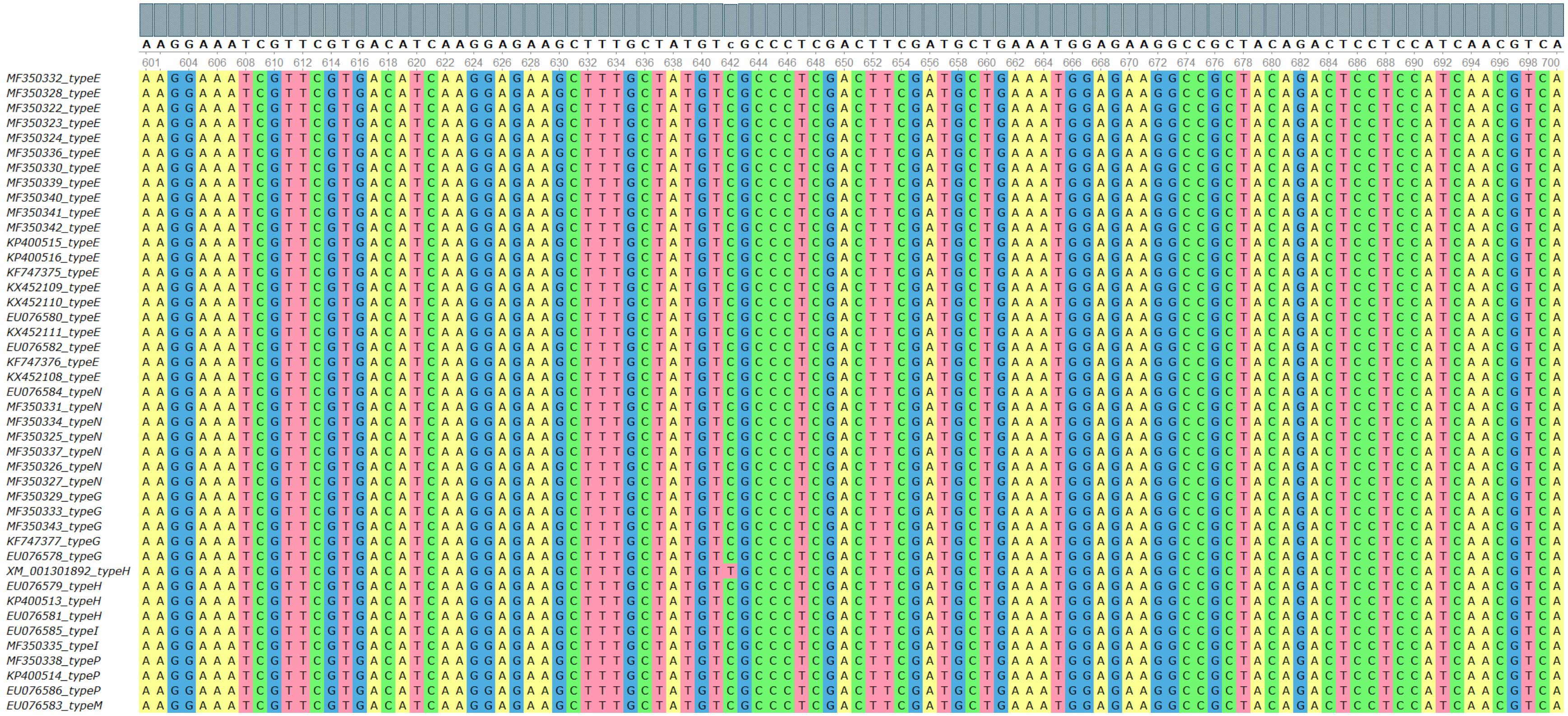
AAGGAAATCGTTCGTGACATCAAGGAGAAGCTTTGCTATGTcGCCCTCGACTTCGATGCTGAAATGGAGAAGGCCGCTACAGACTCCTCCATCAACGTCA
601 604 606 608 610 612 614 616 618 620 622 624 626 628 630 632 634 636 638 640 642 644 646 648 650 652 654 656 658 660 662 664 666 668 670 672 674 676 678 680 682 684 686 688 690 692 694 696 698 700
MF350332_typeE
MF350328_typeE
MF350322_typeE
MF350323_typeE
MF350324_typeE
MF350336_typeE
MF350330_typeE
MF350339_typeE
MF350340_typeE
MF350341_typeE
MF350342_typeE
KP400515_typeE
KP400516_typeE
KF747375_typeE
KX452109_typeE
KX452110_typeE
EU076580_typeE
KX452111_typeE
EU076582_typeE
KF747376_typeE
KX452108_typeE
EU076584_typeN
MF350331_typeN
MF350334_typeN
MF350325_typeN
MF350337_typeN
MF350326_typeN
MF350327_typeN
MF350329_typeG
MF350333_typeG
MF350343_typeG
KF747377_typeG
EU076578_typeG
XM_001301892_typeH
EU076579_typeH
KP400513_typeH
EU076581_typeH
EU076585_typeI
MF350335_typeI
MF350338_typeP
KP400514_typeP
EU076586_typeP
EU076583_typeM

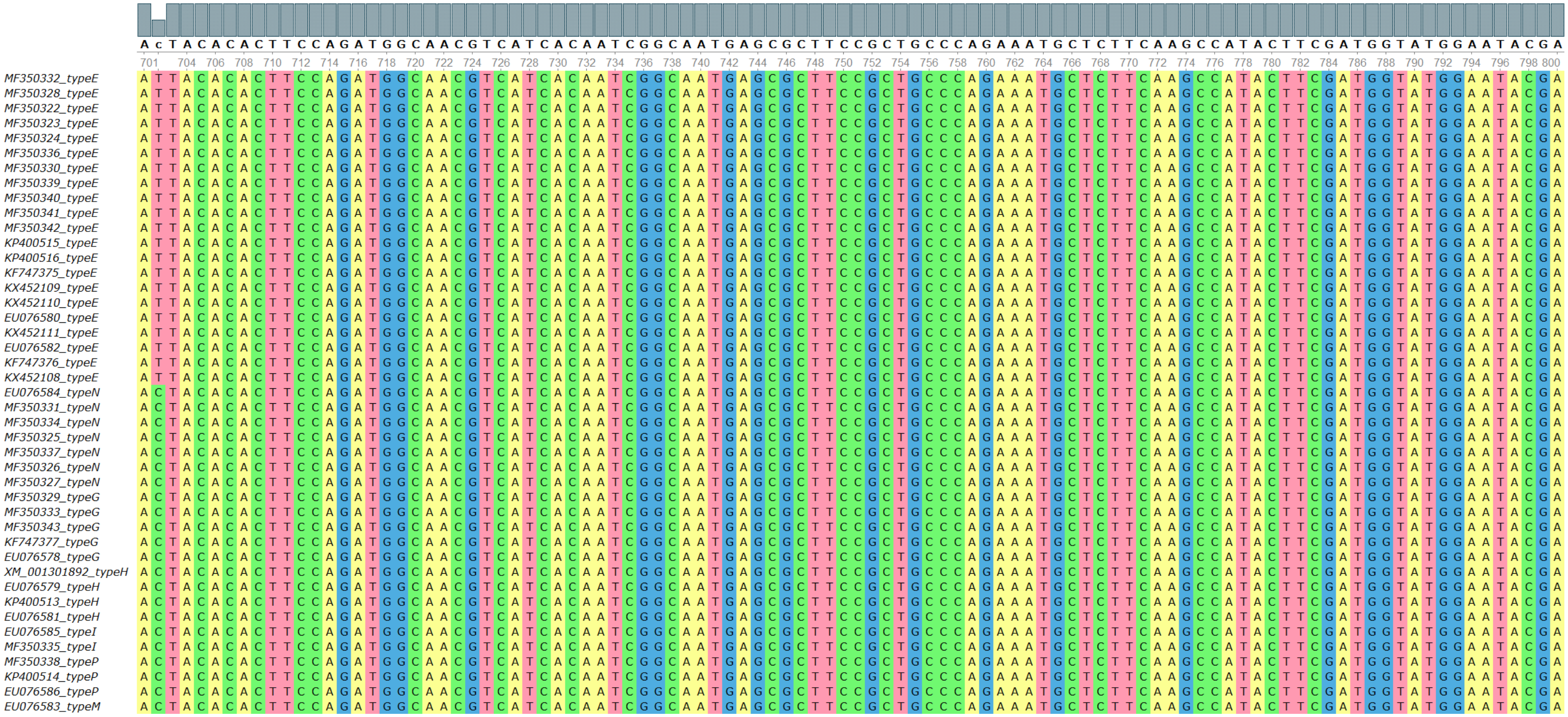
AcTACACACTTCCAGATGGCAACGTCATCACAATCGGCAATGAGCGCTTCCGCTGCCCAGAAATGCTCTTCAAGCCATACTTCGATGGTATGGAATACGA
701 704 706 708 710 712 714 716 718 720 722 724 726 728 730 732 734 736 738 740 742 744 746 748 750 752 754 756 758 760 762 764 766 768 770 772 774 776 778 780 782 784 786 788 790 792 794 796 798 800
MF350332_typeE ATTACACACTTCCAGATGGCAACGTCATCACAATCGGCAATGAGCGCTTCCGCTGCCCAGAAATGCTCTTCAAGCCATACTTCGATGGTATGGAATACGA
MF350328_typeE ATTACACACTTCCAGATGGCAACGTCATCACAATCGGCAATGAGCGCTTCCGCTGCCCAGAAATGCTCTTCAAGCCATACTTCGATGGTATGGAATACGA
MF350322_typeE ATTACACACTTCCAGATGGCAACGTCATCACAATCGGCAATGAGCGCTTCCGCTGCCCAGAAATGCTCTTCAAGCCATACTTCGATGGTATGGAATACGA
MF350323_typeE ATTACACACTTCCAGATGGCAACGTCATCACAATCGGCAATGAGCGCTTCCGCTGCCCAGAAATGCTCTTCAAGCCATACTTCGATGGTATGGAATACGA
MF350324_typeE ATTACACACTTCCAGATGGCAACGTCATCACAATCGGCAATGAGCGCTTCCGCTGCCCAGAAATGCTCTTCAAGCCATACTTCGATGGTATGGAATACGA
MF350336_typeE ATTACACACTTCCAGATGGCAACGTCATCACAATCGGCAATGAGCGCTTCCGCTGCCCAGAAATGCTCTTCAAGCCATACTTCGATGGTATGGAATACGA
MF350330_typeE ATTACACACTTCCAGATGGCAACGTCATCACAATCGGCAATGAGCGCTTCCGCTGCCCAGAAATGCTCTTCAAGCCATACTTCGATGGTATGGAATACGA
MF350339_typeE ATTACACACTTCCAGATGGCAACGTCATCACAATCGGCAATGAGCGCTTCCGCTGCCCAGAAATGCTCTTCAAGCCATACTTCGATGGTATGGAATACGA
MF350340_typeE ATTACACACTTCCAGATGGCAACGTCATCACAATCGGCAATGAGCGCTTCCGCTGCCCAGAAATGCTCTTCAAGCCATACTTCGATGGTATGGAATACGA
MF350341_typeE ATTACACACTTCCAGATGGCAACGTCATCACAATCGGCAATGAGCGCTTCCGCTGCCCAGAAATGCTCTTCAAGCCATACTTCGATGGTATGGAATACGA
MF350342_typeE ATTACACACTTCCAGATGGCAACGTCATCACAATCGGCAATGAGCGCTTCCGCTGCCCAGAAATGCTCTTCAAGCCATACTTCGATGGTATGGAATACGA
KP400515_typeE ATTACACACTTCCAGATGGCAACGTCATCACAATCGGCAATGAGCGCTTCCGCTGCCCAGAAATGCTCTTCAAGCCATACTTCGATGGTATGGAATACGA
KP400516_typeE ATTACACACTTCCAGATGGCAACGTCATCACAATCGGCAATGAGCGCTTCCGCTGCCCAGAAATGCTCTTCAAGCCATACTTCGATGGTATGGAATACGA
KF747375_typeE ATTACACACTTCCAGATGGCAACGTCATCACAATCGGCAATGAGCGCTTCCGCTGCCCAGAAATGCTCTTCAAGCCATACTTCGATGGTATGGAATACGA
KX452109_typeE ATTACACACTTCCAGATGGCAACGTCATCACAATCGGCAATGAGCGCTTCCGCTGCCCAGAAATGCTCTTCAAGCCATACTTCGATGGTATGGAATACGA
KX452110_typeE ATTACACACTTCCAGATGGCAACGTCATCACAATCGGCAATGAGCGCTTCCGCTGCCCAGAAATGCTCTTCAAGCCATACTTCGATGGTATGGAATACGA
EU076580_typeE ATTACACACTTCCAGATGGCAACGTCATCACAATCGGCAATGAGCGCTTCCGCTGCCCAGAAATGCTCTTCAAGCCATACTTCGATGGTATGGAATACGA
KX452111_typeE ATTACACACTTCCAGATGGCAACGTCATCACAATCGGCAATGAGCGCTTCCGCTGCCCAGAAATGCTCTTCAAGCCATACTTCGATGGTATGGAATACGA
EU076582_typeE ATTACACACTTCCAGATGGCAACGTCATCACAATCGGCAATGAGCGCTTCCGCTGCCCAGAAATGCTCTTCAAGCCATACTTCGATGGTATGGAATACGA
KF747376_typeE ATTACACACTTCCAGATGGCAACGTCATCACAATCGGCAATGAGCGCTTCCGCTGCCCAGAAATGCTCTTCAAGCCATACTTCGATGGTATGGAATACGA
KX452108_typeE ATTACACACTTCCAGATGGCAACGTCATCACAATCGGCAATGAGCGCTTCCGCTGCCCAGAAATGCTCTTCAAGCCATACTTCGATGGTATGGAATACGA
EU076584_typeN ACTACACACTTCCAGATGGCAACGTCATCACAATCGGCAATGAGCGCTTCCGCTGCCCAGAAATGCTCTTCAAGCCATACTTCGATGGTATGGAATACGA
MF350331_typeN ACTACACACTTCCAGATGGCAACGTCATCACAATCGGCAATGAGCGCTTCCGCTGCCCAGAAATGCTCTTCAAGCCATACTTCGATGGTATGGAATACGA
MF350334_typeN ACTACACACTTCCAGATGGCAACGTCATCACAATCGGCAATGAGCGCTTCCGCTGCCCAGAAATGCTCTTCAAGCCATACTTCGATGGTATGGAATACGA
MF350325_typeN ACTACACACTTCCAGATGGCAACGTCATCACAATCGGCAATGAGCGCTTCCGCTGCCCAGAAATGCTCTTCAAGCCATACTTCGATGGTATGGAATACGA
MF350337_typeN ACTACACACTTCCAGATGGCAACGTCATCACAATCGGCAATGAGCGCTTCCGCTGCCCAGAAATGCTCTTCAAGCCATACTTCGATGGTATGGAATACGA
MF350326_typeN ACTACACACTTCCAGATGGCAACGTCATCACAATCGGCAATGAGCGCTTCCGCTGCCCAGAAATGCTCTTCAAGCCATACTTCGATGGTATGGAATACGA
MF350327_typeN ACTACACACTTCCAGATGGCAACGTCATCACAATCGGCAATGAGCGCTTCCGCTGCCCAGAAATGCTCTTCAAGCCATACTTCGATGGTATGGAATACGA
MF350329_typeG ACTACACACTTCCAGATGGCAACGTCATCACAATCGGCAATGAGCGCTTCCGCTGCCCAGAAATGCTCTTCAAGCCATACTTCGATGGTATGGAATACGA
MF350333_typeG ACTACACACTTCCAGATGGCAACGTCATCACAATCGGCAATGAGCGCTTCCGCTGCCCAGAAATGCTCTTCAAGCCATACTTCGATGGTATGGAATACGA
MF350343_typeG ACTACACACTTCCAGATGGCAACGTCATCACAATCGGCAATGAGCGCTTCCGCTGCCCAGAAATGCTCTTCAAGCCATACTTCGATGGTATGGAATACGA
KF747377_typeG ACTACACACTTCCAGATGGCAACGTCATCACAATCGGCAATGAGCGCTTCCGCTGCCCAGAAATGCTCTTCAAGCCATACTTCGATGGTATGGAATACGA
EU076578_typeG ACTACACACTTCCAGATGGCAACGTCATCACAATCGGCAATGAGCGCTTCCGCTGCCCAGAAATGCTCTTCAAGCCATACTTCGATGGTATGGAATACGA
XM_001301892_typeH ACTACACACTTCCAGATGGCAACGTCATCACAATCGGCAATGAGCGCTTCCGCTGCCCAGAAATGCTCTTCAAGCCATACTTCGATGGTATGGAATACGA
EU076579_typeH ACTACACACTTCCAGATGGCAACGTCATCACAATCGGCAATGAGCGCTTCCGCTGCCCAGAAATGCTCTTCAAGCCATACTTCGATGGTATGGAATACGA
KP400513_typeH ACTACACACTTCCAGATGGCAACGTCATCACAATCGGCAATGAGCGCTTCCGCTGCCCAGAAATGCTCTTCAAGCCATACTTCGATGGTATGGAATACGA
EU076581_typeH ACTACACACTTCCAGATGGCAACGTCATCACAATCGGCAATGAGCGCTTCCGCTGCCCAGAAATGCTCTTCAAGCCATACTTCGATGGTATGGAATACGA
EU076585_typeI ACTACACACTTCCAGATGGCAACGTCATCACAATCGGCAATGAGCGCTTCCGCTGCCCAGAAATGCTCTTCAAGCCATACTTCGATGGTATGGAATACGA
MF350335_typeI ACTACACACTTCCAGATGGCAACGTCATCACAATCGGCAATGAGCGCTTCCGCTGCCCAGAAATGCTCTTCAAGCCATACTTCGATGGTATGGAATACGA
MF350338_typeP ACTACACACTTCCAGATGGCAACGTCATCACAATCGGCAATGAGCGCTTCCGCTGCCCAGAAATGCTCTTCAAGCCATACTTCGATGGTATGGAATACGA
KP400514_typeP ACTACACACTTCCAGATGGCAACGTCATCACAATCGGCAATGAGCGCTTCCGCTGCCCAGAAATGCTCTTCAAGCCATACTTCGATGGTATGGAATACGA
EU076586_typeP ACTACACACTTCCAGATGGCAACGTCATCACAATCGGCAATGAGCGCTTCCGCTGCCCAGAAATGCTCTTCAAGCCATACTTCGATGGTATGGAATACGA
EU076583_typeM ACTACACACTTCCAGATGGCAACGTCATCACAATCGGCAATGAGCGCTTCCGCTGCCCAGAAATGCTCTTCAAGCCATACTTCGATGGTATGGAATACGA

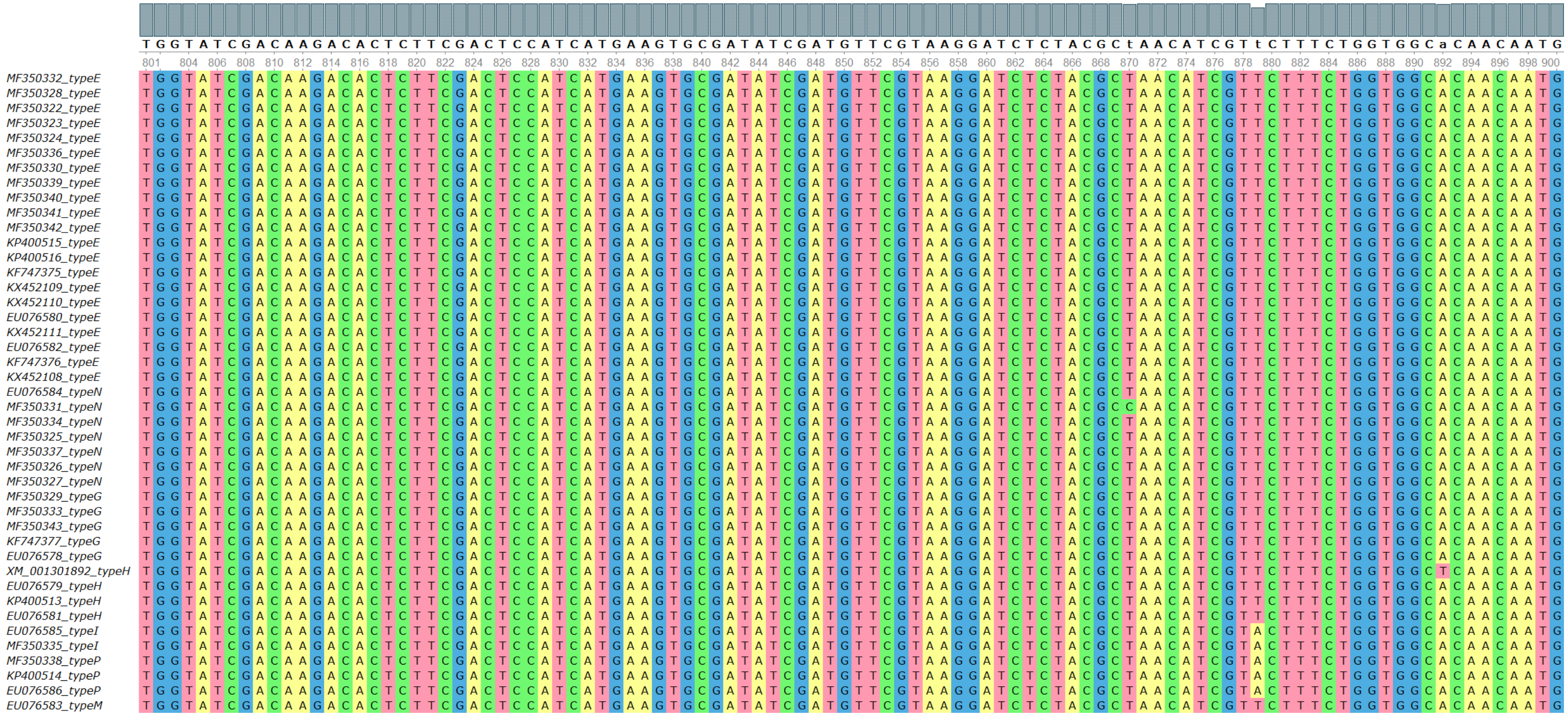
TGGTATCGACAAGACACTCTTCGACTCCATCATGAAGTGCGATATCGATGTTCGTAAGGATCTCTACGCtAACATCGTtCTTTCTGGTGGCaCAACAATG
801 804 806 808 810 812 814 816 818 820 822 824 826 828 830 832 834 836 838 840 842 844 846 848 850 852 854 856 858 860 862 864 866 868 870 872 874 876 878 880 882 884 886 888 890 892 894 896 898 900
MF350332_typeE
MF350328_typeE
MF350322_typeE
MF350323_typeE
MF350324_typeE
MF350336_typeE
MF350330_typeE
MF350339_typeE
MF350340_typeE
MF350341_typeE
MF350342_typeE
KP400515_typeE
KP400516_typeE
KF747375_typeE
KX452109_typeE
KX452110_typeE
EU076580_typeE
KX452111_typeE
EU076582_typeE
KF747376_typeE
KX452108_typeE
EU076584_typeN
MF350331_typeN
MF350334_typeN
MF350325_typeN
MF350337_typeN
MF350326_typeN
MF350327_typeN
MF350329_typeG
MF350333_typeG
MF350343_typeG
KF747377_typeG
EU076578_typeG
XM_001301892_typeH
EU076579_typeH
KP400513_typeH
EU076581_typeH
EU076585_typeI
MF350335_typeI
MF350338_typeP
KP400514_typeP
EU076586_typeP
EU076583_typeM

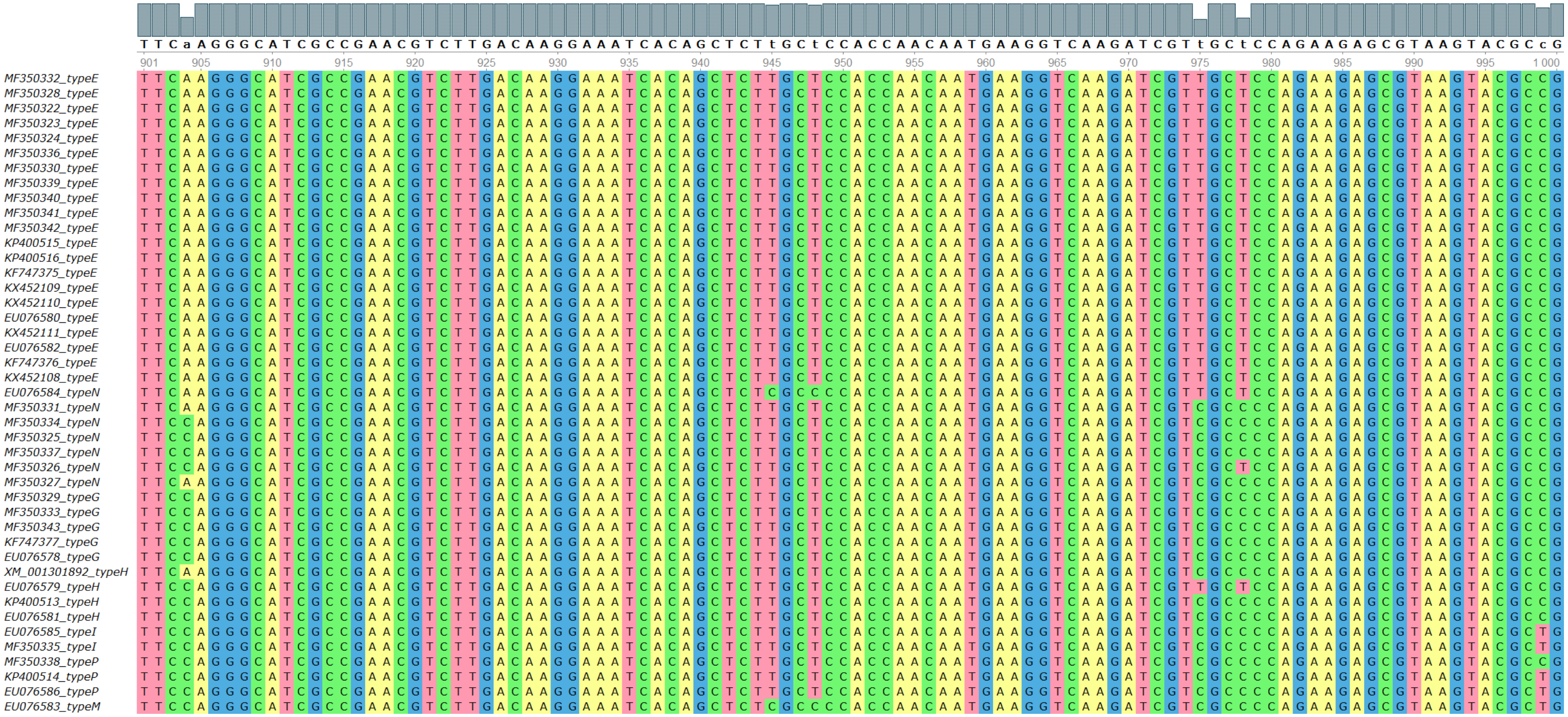
TTCaAGGGCATCGCCGAACGTCTTGACAAGGAAATCACAGCTCTtGCtCCACCAACAATGAAGGTCAAGATCGTtGCtCCAGAAGAGCGTAAGTACGCcG
901 905 910 915 920 925 930 935 940 945 950 955 960 965 970 975 980 985 990 995 1 000
MF350332_typeE
MF350328_typeE
MF350322_typeE
MF350323_typeE
MF350324_typeE
MF350336_typeE
MF350330_typeE
MF350339_typeE
MF350340_typeE
MF350341_typeE
MF350342_typeE
KP400515_typeE
KP400516_typeE
KF747375_typeE
KX452109_typeE
KX452110_typeE
EU076580_typeE
KX452111_typeE
EU076582_typeE
KF747376_typeE
KX452108_typeE
EU076584_typeN
MF350331_typeN
MF350334_typeN
MF350325_typeN
MF350337_typeN
MF350326_typeN
MF350327_typeN
MF350329_typeG
MF350333_typeG
MF350343_typeG
KF747377_typeG
EU076578_typeG
XM_001301892_typeH
EU076579_typeH
KP400513_typeH
EU076581_typeH
EU076585_typeI
MF350335_typeI
MF350338_typeP
KP400514_typeP
EU076586_typeP
EU076583_typeM

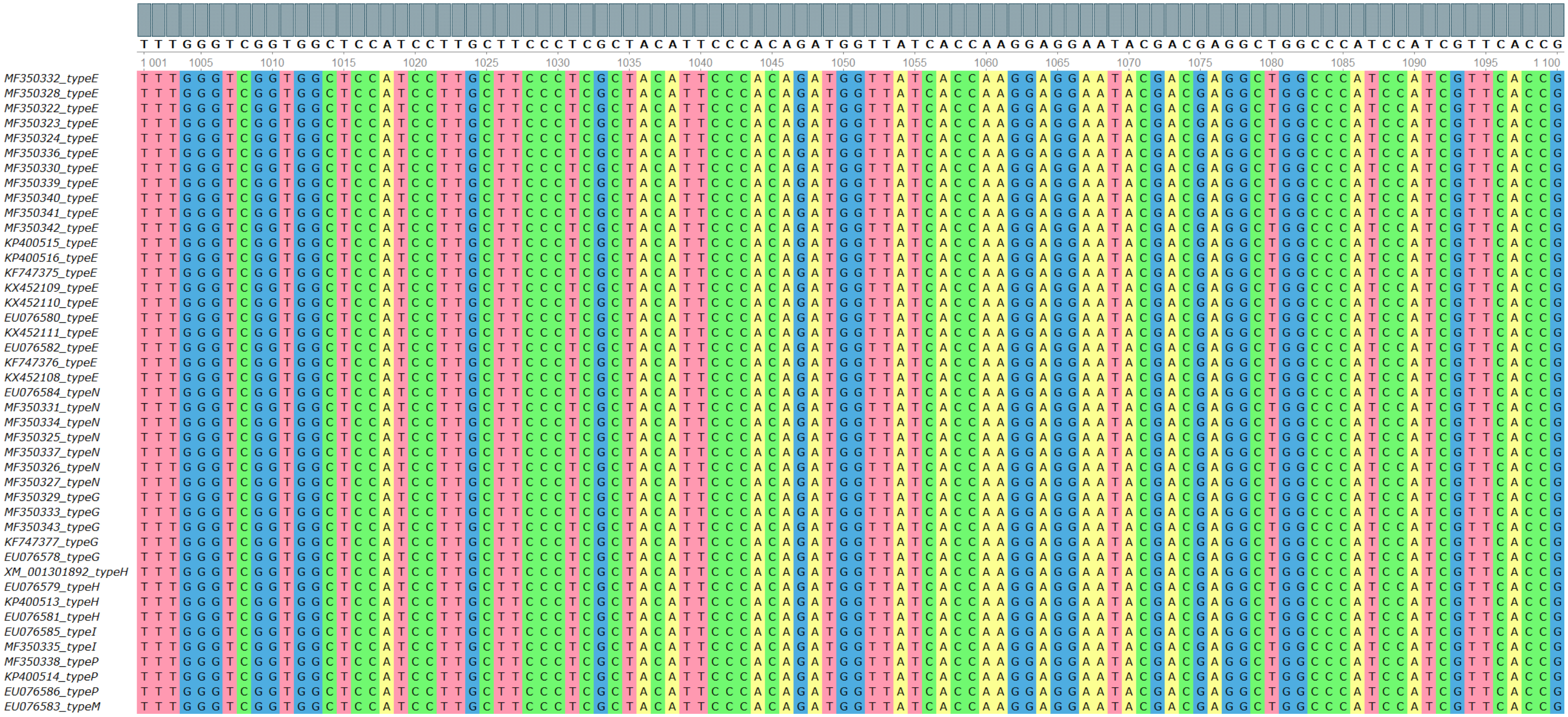

Sequences accession numbers with the prefix MF are from our study, the other *T. vaginalis actin* sequences were retrieved from GenBank, and the suffix letter indicates the assigned *actin* genotype.
